# Inhibition of RORγt activity and Th17 differentiation by a set of novel compounds

**DOI:** 10.1186/s12865-015-0097-9

**Published:** 2015-05-29

**Authors:** Qingfeng Ding, Mei Zhao, Chuan Bai, Bolan Yu, Zhaofeng Huang

**Affiliations:** Institute of Human Virology, Zhongshan School of Medicine, Sun Yat-sen University, N1311 Rm, No.10 Bld, 74 Zhongshan 2nd Rd, Guangzhou, 510080 China; Key Laboratory of Tropical Diseases Control, Ministry of Education in China, Sun Yat-sen University, Guangzhou, China; Key Laboratory for Major Obstetric Diseases of Guangdong Province, Third Affiliated Hospital of Guangzhou Medical University, Guangzhou, China; Department of Biochemistry, Zhongshan School of Medicine, Sun Yat-sen University, Guangzhou, China

**Keywords:** RORγt, Th17 cell, Autoimmune disease, IL-17A

## Abstract

**Background:**

Retinoic acid receptor-related orphan receptor gamma t (RORγt) is the master regulator of Th17 cell differentiation, which plays a critical role in the pathology of several autoimmune diseases. By directing Th17 cells function, RORγt could be a potential target for drug development for Th17 related autoimmune disease.

**Methods:**

A Jurkat cell-based reporter assay system was used for screening RORγt inhibitors from a drug-like chemical library, following with mouse Th17 cells differentiation study to identify the effect of targeted compounds in primary T cells. 293T cell-based reporter assay was conducted to determine the cell specificity, and MTT assay was performed to determine the cell toxicity of those compounds.

**Results:**

In this study, we identified four lead compounds that suppressed RORγt activity, Th17 differentiation and IL-17A secretion. These candidates displayed inhibition ability on RORγt activity in T cell derived Jurkat cell, but not in 293 T cell, which indicated the restricted effects of these compounds to other cells or tissues. Futhermore, our results demonstrated that these candidates exhibited more robust inhibitory on IL-17 F transcription expression than IL-17A, which is different from one reported compound, SR1001, that mainly suppressed IL-17A, rather than IL-17 F production.

**Conclusions:**

Our study discovered four novel compounds that inhibited RORγt activity and Th17 function, which indicates their potential in therapeutic application of Th17 related autoimmune disorders.

## Background

The retinoic acid receptor-related orphan gamma t (RORγt) is a member of the nuclear receptor superfamily that plays a key role in autoimmune disease [1, 2]. Two distinct RORγ isoforms exist in mice (RORγ1 and RORγ2), which vary in their N-terminal mRNA sequences [3]. Human RORγt (also known as RORγ2) mRNA was first isolated from human pancreas, and the protein is comprised of several conserved domains. Studies revealed that RORγt expression is specific to certain T cell compartments, which indicated its critical role in T cell development [4, 5].

In 2006, Ivanov et al. discovered the direct linkage between RORγt signaling and Th17 differentiation in the periphery [4]. It was later determined that RORγt also promotes IL-17A production and Th17 polarization [6]. Th17 cells have been implicated in the pathogenesis of autoimmune diseases—such as multiple sclerosis (MS) and rheumatoid arthritis (RA) [7–10], as well as in chronic inflammation in both human and an experimental mouse model of autoimmune encephalomyelitis (EAE) [11, 12]. Together with IL-17A, Th17 cells also secrete another proinflammatory cytokine of the IL-17 family, IL-17 F [13]. Increased levels of IL-17 F have also been detected in autoimmunity, suggesting that IL-17 F may play a similar role to that of IL-17A [13]. Recently, studies have elucidated a potential treatment for autoimmune diseases by suppressing IL-17A and IL-17 F production using RORγt inhibitors [14, 15].

Structural analysis revealed that RORγt contains a variable N-terminal domain, DNA-binding domain (DBD), hinge region, and a C-terminal ligand-binding domain (LBD) [16, 17]. The LBD includes 12 helices and a short β-sheet. Helix 12 in the LBD contains a transactivation function 2 (AF2) domain crucial for genes’ transcriptional activation [16]. AF2 can interact with both coactivators and corepressors to regulate target gene expression and physiological function in vivo [16, 18], which is inhibited by a mutation within this domain [18, 19]. Digoxin and SR1001, as well as its derivatives such as SR1555, have been reported to be chemical ligands capable of modulating RORγt gene transcription by interfering with the active LBD conformation [20–24]. However, digoxin displays dramatic adverse drug reactions with non-specific inhibitions [20, 21], and SR1555 acts as a moderately efficacious inverse agonist with an EC50 value of 1.5 μM in a GAL4 /RORγt cotransfection assay [22–25].

In order to develop more RORγt inhibitors for autoimmune disease therapy, we generated a GAL4/RORγt-LBD cotransfected stable cell lines, and screened a commercial chemical library in this study. We identified four compounds with robust inhibition on RORγt activity as well as on the expression of IL-17A and IL-17 F, indicating the potential utility of these compounds in the therapy of autoimmune diseases.

## Methods

### Plasmids

The pGL4.31 (luc2P/GAL4UAS/Hygro) and pBIND vectors were obtained from Promega (Madison, WI, USA). An IRES-GFP sequence was first cloned into the pBIND vector. Human RORγt was then amplified using cDNA isolated from human peripheral blood mononuclear cells (PBMCs) (provided by Dr. Hui Zhang) and inserted into the reconstructed pBIND-IRES-GFP vector to generate a Gal4–RORγt-LBD-IRES-GFP fusion sequence. The primers that used to construct the pBIND- Gal4–RORγt-LBD-IRES-GFP plasmid are as follows: RORγt-LBD forward 5′- AACTAGGATCCGAAACCGATGCCAGCACTGC-3′,reverse5′- AACTAGGATCCGCCTGCTGACAGAAAGCCA -3′.

### Cell culture

HEK 293 T cells were maintained in DMEM high-glucose medium (Invitrogen, Carlsbad, CA, USA) supplemented with 10 % fetal bovine serum (FBS; Hyclone, Logan, UT, USA) and 100 U/mL penicillin/streptomycin. Jurkat and mouse splenocytes cells were cultured in RPMI1640 medium (Invitrogen) supplemented with 10 % FBS, 2 mM glutamine, 1 mM sodium pyruvate, 50 μM 2-mercaptoethanol, and 100 U/mL penicillin/streptomycin. All cells were incubated at 37 °C in a humidified atmosphere with 5 % CO_2_.

### Establishment of the RORγt-LBD 293 T and Jurkat reporter cell lines

The pGL4.31 reporter plasmid (4 μg) was transfected into 293 T cells (1 × 10^6^) with Lipofectamine™ 2000 (Invitrogen) according to manufacturer instructions. The transfected cells were then selected in a medium containing 100 μg/mL hygromycin B (Roche, San Francisco, CA, USA) for 3 weeks to obtain a cell population that expressed the pGL4.31 reporter gene. Then, the stable cells were transfected with pBIND-RORγt-LBD-IRES-GFP plasmid (4 μg). The cotransfectants were sorted by green fluorescent protein (GFP) expression, which yielded a RORγt-LBD^+^ 293Tstable reporter cell line with >96 % purity as determined by flow cytometry.

A similar procedure was used to generate the Jurkat RORγt -LBD reporter cell line. For this, Jurkat cells (1 × 10^7^) were transfected with pGL4.31 reporter plasmid (20 μg) by electroporation (Lonza, Basel, Switzerland) according to the manufacturer’s instructions. The transfected Jurkat cells were selected in RPMI 1640 medium containing 200 μg/mL hygromycin B prior to a second transfection. Stable Jurkat cotransfectants were then sorted based on GFP expression, and yielded a GFP^+^ purity >96 % as determined by flow cytometry.

### High-throughput screen

RORγt-LBD^**+**^-Jurkat (2 × 10^4^) stable cells were seeded into 96-well round-bottom plates (Corning Inc, Corning, NY) and incubated in RPMI1640 complete medium overnight. Then, Screened compounds (Enamine, Monmouth Junction, NJ) were added to the wells using a Tecan Freedom EVO 150 (Tecan, Männedorf, Switzerland) to a final concentration of 50 μM. Cells were treated with DMSO or 100 nM PMA (Sigma-Aldrich, St. Louis, MO) only for use as negative and positive controls, respectively. After a 6 h treatment period, cells were lysed and assayed for luciferase activity (Promega) following the manufacturer’s instructions.

### Varied inhibition in RORγt-LBD ^+^ -293 T stable cells of the candidates

RORγt-LBD^**+**^-293 T (1x10^4^) stable cells wereseeded into 96-well plates (Corning Inc., Corning, NY)and incubated in DMEM complete medium overnight. Then the candidates were added with a final concentration of 5 μM. Negative control (DMSO) and positive control were carried on? as HTS. 6 h later, the cells were lysed and assayed for luciferase activity (Promega, USA) following the manufacturer’s instructions.

### Mice

C57BL/6 mice, purchased from the Laboratory Animal Center of Sun Yat-Sen University (Guangzhou, China), were maintained in a specific pathogen-free environment in accordance with institutional protocol. All procedures were reviewed and approved by the Ethics Committee of the Zhongshan School of Medicine, Sun Yat-Sen University.

### In vitro T cell differentiation

CD4^+^CD25^−^ T cells were isolated from spleens of 8–12-week-old C57BL/6 mice as follows: single-cell suspensions were made by crushing the spleen through a cell strainer, and red blood cells (RBCs) were lysed with RBC lysis buffer. CD4^+^ T cells were then purified using magnetic-activated cell sorting (MACS) column a CD4^+^ T cell isolation kit (Miltenyi Biotec, Bergisch Gladbach, Germany) according to the manufacturer’s protocol.

Spleen cells (3 × 10^6^) were stimulated in 6-well plates with 5 μg/mL plate-bound anti-CD3e antibody (eBioscience, San Diego, USA) and 2 μg/mL soluble anti-CD28 antibody (eBioscience) for 24 h. The activated cells were then collected, and seeded into 96-well round-bottom plates (2 × 10^4^) for Th17 differentiation in the presence of 5 ng/mL recombinant human TGF-β (R&D Systems, Minneapolis, MN), 30 ng/mL recombinant mouse IL-6 (R&D Systems, Minneapolis, MN), and either the screening compounds (5 μM final concentration) or DMSO as control. After culturing for 2 days, fresh medium (100 uL/well) containing recombinant human TGF-β, recombinant mouse IL-6, and the previously-introduced screening compounds was added to the cells, which were then cultured for additional 2 days [26].

### CDNA synthesis and quantitative PCR

Total RNA was extracted from splenocytes with TRIzol (Invitrogen) after 5 days in culture. Approximately 1 μg total RNA was added to a 20 μL reaction volume for reverse transcription using the GoScript™ Reverse Transcription System (Promega) according to the manufacturer’s instructions. Synthesized cDNA was then used for real-time quantitative PCR using GoTaq® qPCR Master Mix (Promega). Gene expression of mouse *Rorc, Il17a,* and *Il17f* was normalized to that of *Gapdh*. The following primer sequences were used: *Rorc* forward 5′-TGTAATGTGG CCTACTCCTGCA-3′, reverse 5′-AAACTTGACAGCATCTCGGGA-3′; *Il17a* forward 5′- CTCCAGAAGGCCCTCAGACTAC-3′, reverse 5′-AGCTTTCCCTCCGCATTGACACAG -3′; *Il17f* forward 5′-GAGGATAACACTGTGAGAGTTGAC-3′, reverse 5′- GAGTTCATGGTGCTGTCTTCC-3′; *Gapdh* forward 5′-TGGTGAAGGTCGGTGTGAAC-3′, reverse 5′-CCATGTAGTTGAGGTCAATGAAGG-3′.

### Enzyme-linked immunosorbent assay (ELISA)

The concentration of IL-17A in the splenocyte supernatant after 5 days in culture was determined using an ELISA kit (CUSABIO, Wuhan, China) according to the manufacturer’s protocol.

### EC_50_ assay

RORγt-LBD^**+**^-Jurkat cells were seeded into 96-well round-bottom plates (2 × 10^4^) and cultured overnight prior to incubation with the screening compounds at 5-fold gradient final concentrations ranging from 5 μM to 8 nM. Cells were lysed 6 h later and assayed for luciferase activity (Promega) using the manufacturer’s instructions. Additionally, the results from the high-throughput screening at 50 μM concentration of candidates were also applied in the calculation of the EC_50_. The EC_50_ values were determined by plotting the logarithm of compound concentration versus relative luciferase activity to determine the half-maximal effective concentration (EC_50_).

### Cell viability assays

Jurkat cell viability after culturing in the presence of the screened compounds was assessed by MTT assay [27]. In brief, wild type Jurkat cells were seeded (2 × 10^4^) and incubated with the compounds in 5-fold gradient dilutions. MTT (dimethylthiazolyl-2-5-diphenyltetrazolium bromide) dye solution (Sigma, St. Louis, MO, USA) was added 48 h later and incubated at 37 °C for 4 h, MTT was reduced by live cells into a colored formazan product. After centrifugation at 1500 rpm for 5 min, the supernatant was discarded and 100 μL DMSO was added to the plate, which was gently shaken for 10 min to dissolve the formazan product. Absorbance at 570 nm wavelength was recorded using Thermo Scientific Multiskan FC (Thermo Scientific, Waltham, MA, USA). Each treatment was repeated in quadruplicate. Cell viabilities were defined relative to control cells treated only with DMSO, with results used as evaluation of cytotoxicity of candidates. The half-maximal cytotoxic concentration (CC_50_) for each compound was calculated from the dose–response curves with the aid of GraphPad Prism software 5.0 (GraphPad Software, San Diego, CA).

### Statistical analysis

All data are shown as the means ± SEM. Statistical significance was determined by an unpaired *t* test using GraphPad Prism software 5.0 (GraphPad Software, San Diego, CA). P values <0.05 were considered to be statistically significant.

## Results

### Validation of the RORγt-LBD^+^ Jurkat stable reporter cell lines

**Fig. 1:**
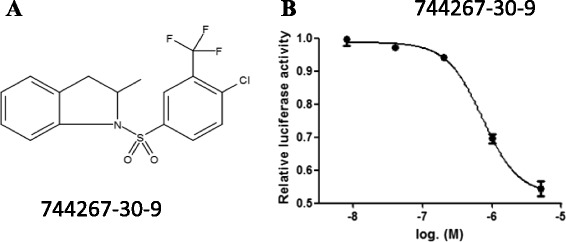
Validation of the RORγt-LBD^+^ Jurkat stable cell line. RORγt-LBD^+^ Jurkat cells were seeded into 96-well round-bottom plates (2 × 10^4^) and cultured overnight prior to incubation with the positive control compound (CAS registry number is 744267-30-9; 1**a**) at 5-fold gradient dilutions with final concentrations ranging from 5 μM to 8 nM for 6 h. Cells were lysed 6 h later and assayed for luciferase activity. The EC_50_ value of the 744267-30-9 was 723nM based on the relative luciferase activity (1**b**). The results were shown as the means ± SEM

Studies have reported that T0901317 is a potent and efficacious agonist of RORγt. We have found that 1- [4- chloro- 3- (trifluoromethyl) phenyl] sulfonyl- 2- methyl- 2, 3- dihydroindole (CAS registry number is 744267-30-9; Fig. 1a), which is structurally similar to T0901317 [22], also suppresses the activity of RORγt, and inhibits IL-17A and IL-17 F production as well as Th17 differentiation (data not published). To validate the RORγt reporter system, we used 744267-30-9 as positive control to assess the change of luciferase activity in the RORγt-LBD^+^ Jurkat reporter cells. For this, RORγt-LBD^+^ Jurkat cells were seeded into 96-well round-bottom plates (2 × 10^4^) and cultured overnight prior to the addition of 5-fold gradient dilutions of 744267-30-9, which ranged from 5 μM to 8 nM. The EC_50_ value of 744267-30-9 with the Gal4-reporter system was 723 nM and confirmed that these cells could be used to evaluate the activity of RORγt inhibitors (Fig. 1b).

### Candidate identification by high-throughput screening (HTS)

After validation, we developed a high-throughput screening (HTS) system using the RORγt-LBD^+^ Jurkat cells to test the activity of RORγt antagonists, which was then used to screen a commercially-available drug-like chemical library. For this, RORγt-LBD^+^ Jurkat cells were treated with the compounds at a final concentration of 50 μM for 6 h, at which point the luciferase reporter activity was measured. Compounds showing inhibitory activity at 50 μM (primary hits) were then challenged at 5 μM concentration for 6 h in a secondary screen. The HTS identified 36 candidate compounds that were likely to inhibit RORγt function.

### Novel compounds inhibit mouse Th17 cell differentiation

Since RORγt activity is essential for optimal Th17 cell development, we further explored whether these compounds could sufficiently inhibit Th17 differentiation in vitro. After stimulated 1 day, naïve mouse CD4^+^ T cells were cultured with IL-6 and TGF-β to induce Th17-polarizing conditions in the presence of the candidate compound (5 μM) or vehicle control (DMSO) for another 4 days.

**Fig. 2:**
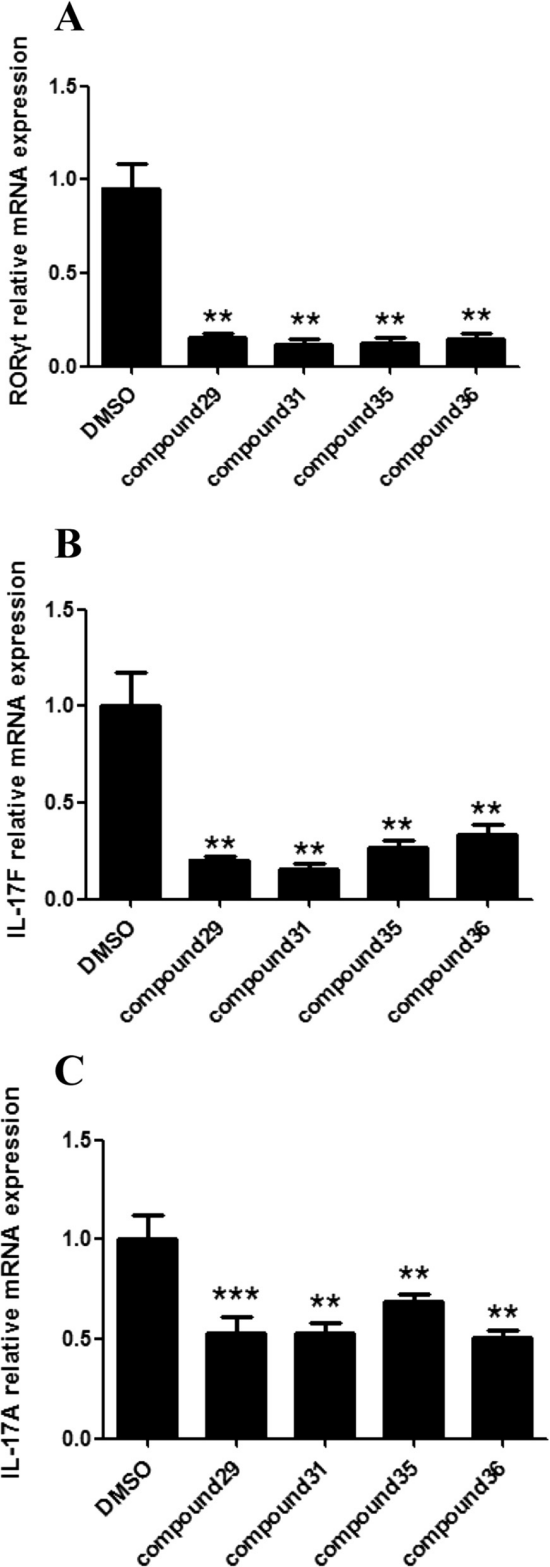
Four screened compounds inhibit mouse Th17 cell differentiation. CD4^+^CD25^−^ T cells were isolated from the spleens of 8–12-week-old mice and cultured under Th17-polarizing conditions in the presence of one of the screened candidate compounds (5 μM) or DMSO for 4 days. RORγt (2**a**), IL-17 F (2**b**), and IL-17 F (2**c**) mRNA expression was quantified and normalized to GAPDH. The results were shown as the means ± SEM. ** P < 0.005

As expected, the combination of TGF-β and IL-6 increased the expression of *Il17a*, *Il17f*, and *RORγt* mRNA in vehicle-treated cells. All 36 compounds had a noticeably suppressive effect on the *RORγt* transcription expression (Fig. 2a), whereas only four (compound 29, compound 31, compound 35, and compound 36) were able to significantly reduce *Il17f* and *Il17a* gene expression, respectively, compared with vehicle-treated control group (Fig. 2b, c; compounds without inhibitory effects are not shown), demonstrated strong inhibition on Th17 cell differentiation and function. Structures of these compounds were summarized in Table 1.

**Table 1:** The structures of compound 29, 31, 35 and 36

Compound	Name	Structure
compound29	3- (5- methyl- 1, 2- oxazol- 3- yl) - 1- [(4- propan- 2- ylphenyl) - thiophen- 2- ylmethyl] urea	
compound31	4- cyclohexyl- N- (1, 1- dioxothiolan- 3- yl) - N- (oxolan- 2- ylmethyl) benzamide	
compound35	1- (3, 4- dimethylphenyl) sulfonyl- N- (5- ethyl- 1, 3, 4- thiadiazol- 2- yl) cyclopentane- 1- carboxamide	
compound36	2- Thiophenecarboxylic acid, 5- methyl- 4- propyl-,2- [[(cyclopentylamino) carbonyl] amino] - 2- oxoethyl ester	

### Candidate compounds inhibit IL-17A secretion

**Fig. 3:**
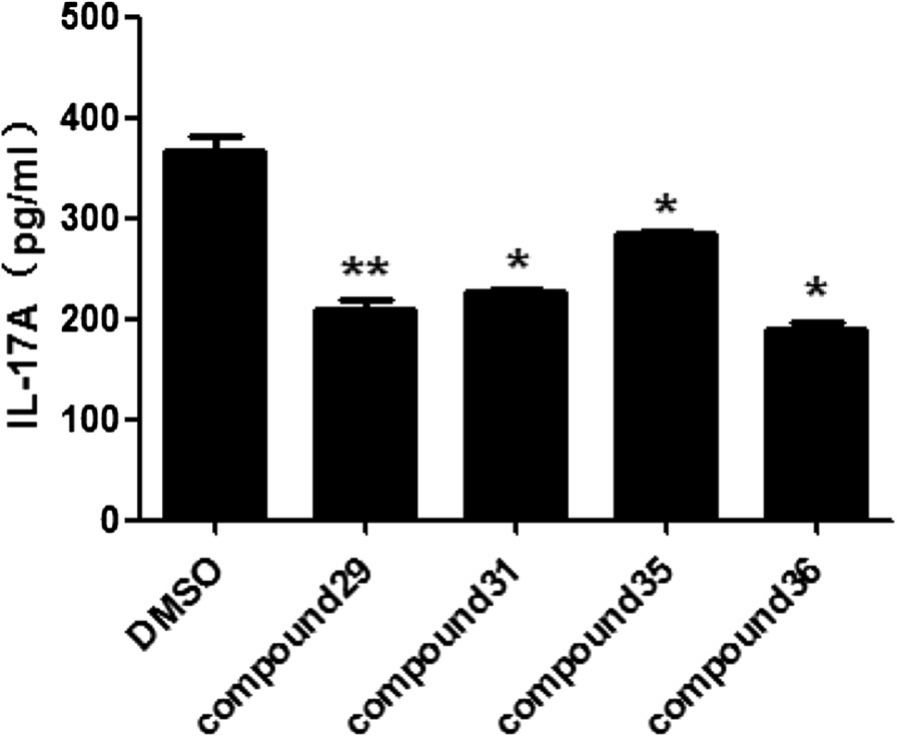
Candidate compounds inhibit IL-17A secretion. Splenocytes were cultured under Th17-polarizing conditions in the presence of the noted compounds. The concentrations of IL-17A in the culture supernatants were then determined by enzyme-linked immunosorbent assay (ELISA). The results were shown as means ± SEM; * P < 0.05; ** P < 0.005

Naïve mouse CD4^+^ T cells were cultured for 5 days, then the culture supernatant was collected and the concentration of IL-17A in the culture supernatant was measured with ELISA assay. In accordance with our mRNA expression data, treatment with these four compounds also suppressed IL-17A secretion. IL-17A concentration from cells treated by compounds 29, 31, 35, and 36 was 209 pg/mL, 226 pg/mL, 284 pg/mL, 189 pg/mL with reductions of 44 %, 39 %, 33 %, and 49 %, respectively, compared with vehicle-treated control cells (Fig. 3).

### Gal4/RORγt EC_50_ values of candidate compounds

To determine the EC_50_ for each compound with respect to our Gal4/RORγt-reporter system, RORγt-LBD^+^ Jurkat cells were treated with increasing concentrations of the candidate compounds for 6 h and the relative luciferase activity was recorded to obtain EC_50_ values.

**Fig. 4:**
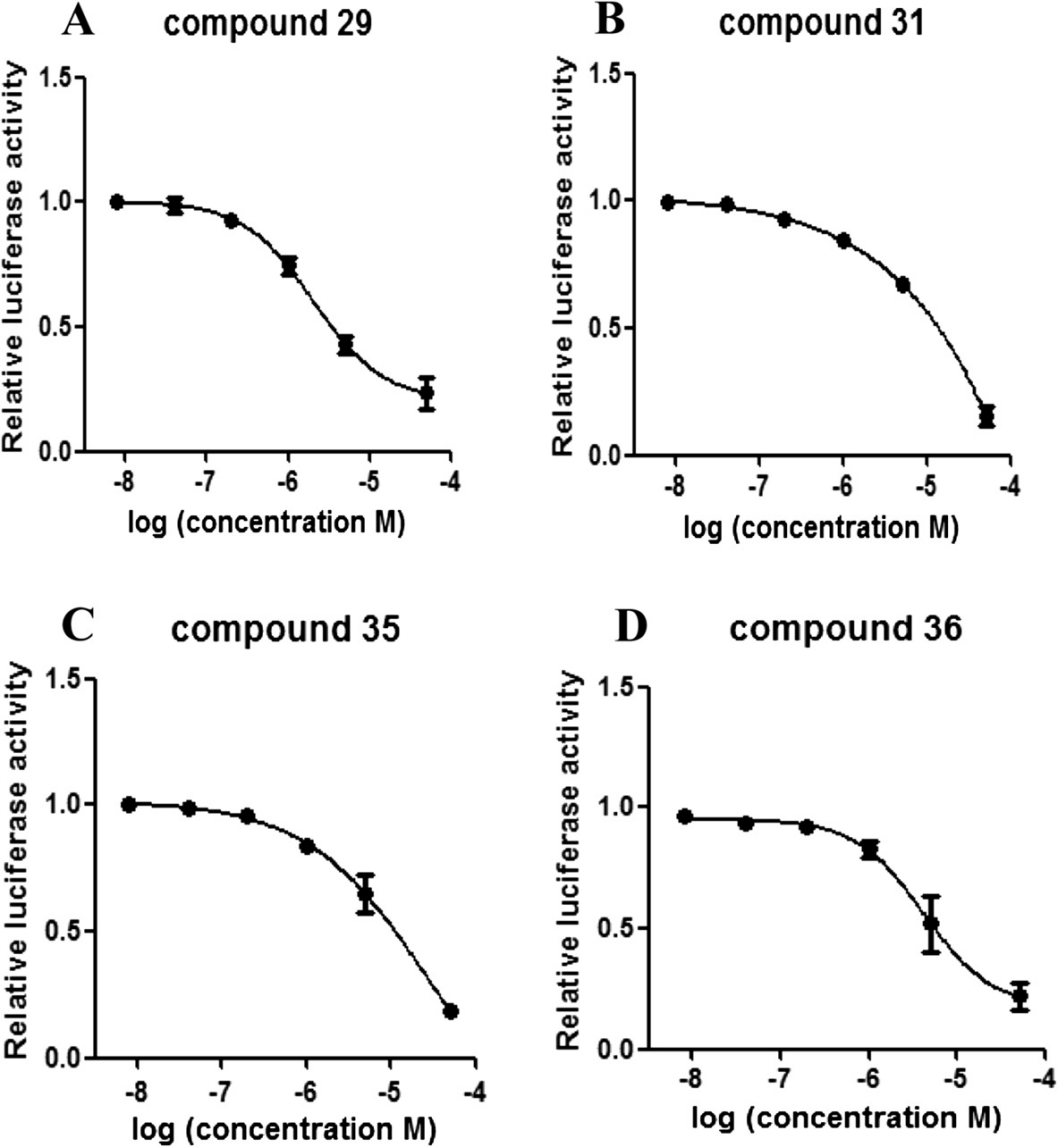
Gal4/RORγt EC_50_ values of candidate compounds. **a**, **b**, **c**, and **d** show the EC_50_ values of compound 29, compound 31, compound 35, and compound 36 respectively. RORγt-LBD^+^ Jurkat cells were seeded into 96-well round-bottom plates (2 × 10^4^) and cultured overnight prior to incubation with increasing concentrations of the candidate compounds for 6 h. Cells were lysed and the relative luciferase activity was recorded to calculate the half-maximal effective concentrations (EC_50_). The results were shown as means ± SEM

In the reporter system, compound 31 and compound 35 displayed weaker inhibition on RORγt activity where the EC_50_ values were 17.46 μM and 28 μM, respectively (Fig. 4b and c). Differing from these, compound 29 and compound 36 exhibited potently inhibitory effects, with EC_50_ values of 2.1 μM and 4.2 μM, respectively (Fig. 4a and d). These results indicated that the two candidates have moderate efficacies in suppressing RORγt activity in our reporter system.

### Inhibition of RORγt activity in 293 T cells

**Fig. 5:**
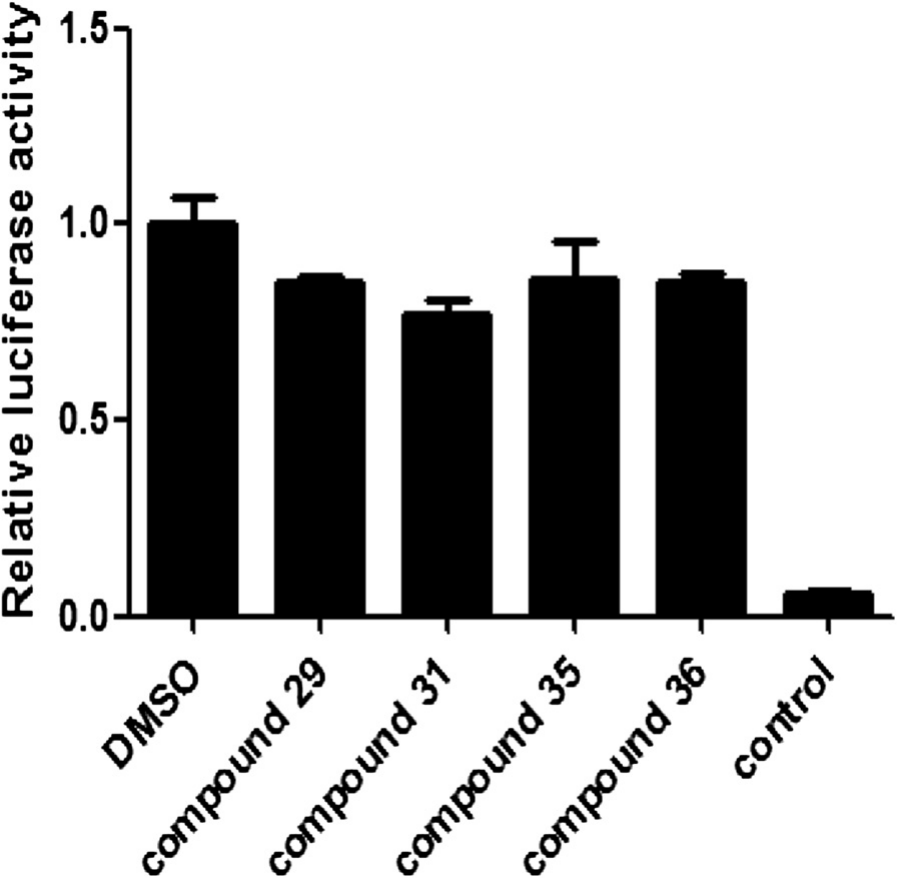
Inhibition of RORγt activity in 293 T cells. RORγt-LBD^+^ 293 T stable cells were cultured in the presence of the candidate compounds (5 μM) and DMSO (vehicle control) for 6 h and the Luciferase reporter activities were then recorded. The results were shown as the means ± SEM

RORγt-LBD^+^-293 T cells were employed to test these compounds’ inhibition in different cell types. Results showed that four compounds slightly inhibited RORγt activity in 293 T cells with less than 20 % reduction at 5uM treatment (Fig. 5). Obviously, these results were lower than those in Jurkat counterparts. In contrast, the positive control compound exhibited strongly inhibited activity in RORγt-LBD^+^-293 T cells similar to thosein RORγt-LBD^+^-Jurkat cells. These results suggest that these four candidate compounds could inhibit RORγt activity in T cell specific pattern.

### CC_50_ values of the candidate compounds

**Fig. 6:**
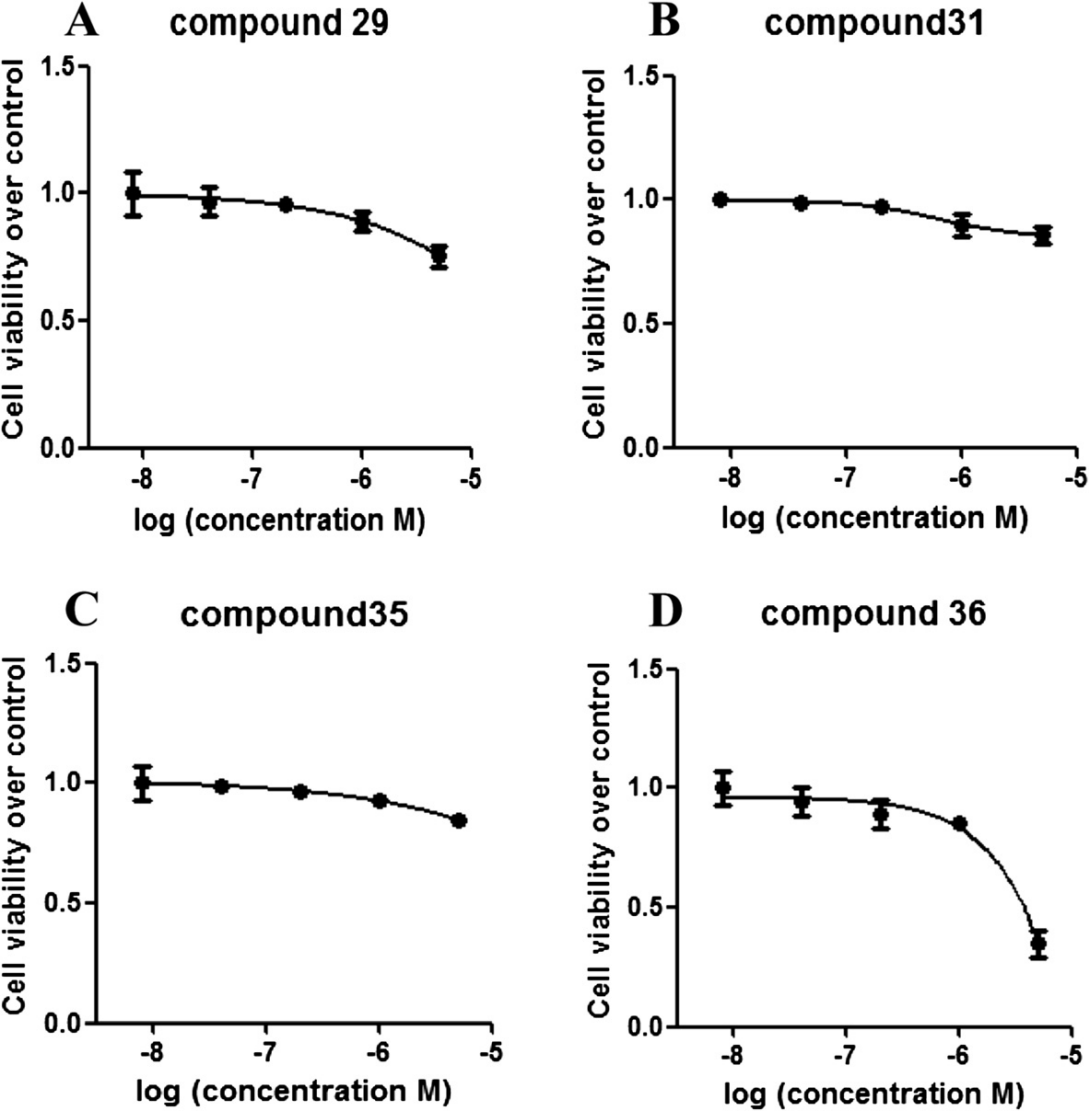
CC_50_ values of novel candidate compounds. **a**, **b**, **c**, and **d** represent CC_50_ values for compound 29, compound 31, compound 35, and compound 36, respectively. Wild-type Jurkat cells were seeded into 96-well round-bottom plates (2 × 10^4^) and cultured overnight prior to incubation with increasing concentrations of these compounds for 48 h. MTT assays were performed and the absorbance at 570 nm was recorded to obtain the CC_50_ value for each compound. The results were shown as means ± SEM

To determine the effect of the candidate compounds on cell viability and cell toxicity, Jurkat cells were cultured with increasing concentrations of these compounds for 48 h and MTT assays were performed to obtain CC_50_ values (Fig. 6). Results displayed that compounds 29, 31, and 35 slightly inhibited Jurkat cell viability with less than 25 % reduction over vehicle control at 5 μM (the highest concentration in this assay) (Fig. 6a, b, c). Although compound 36 showed slightly higher cytotoxicity with about 50 % reduction at 5 μM (Fig. 6d), CC_50_ values of these four candidates all are higher than 5 μM (the highest concentration in this assay), indicated low toxicity of these compounds in Jurkat cells.

## Discussion

RORγt is essential for both IL-17A and IL-17 F production, as well as the differentiation of Th17 cells [28]. Th17 cells have implications for mediating the pathology of several autoimmune disorders in both humans and mice [8–11, 29]. Recently, several studies have reported on the development of novel therapeutics to treat autoimmune disease by inhibiting the activity of RORγt by exogenous chemical ligands [20–24]. Among these studies, SR1001 and its several derivatives have been considered ideal candidates [22–25]. In this study, we screened a chemical library and identified four novel lead compounds (compound 29, compound 31, compound 35, and compound 36) that effectively suppress RORγt activity and Th17 differentiation.

RORγt, which belong to the nuclear receptors superfamily, is highly expressed in thymus, liver, brain, pancreas, muscle and other organs, and is involved in many physiological processes [30–32]. Beside T cell related functions, RORγt is also involved in the regulation of lipid and glucose metabolism, phase I and phase II enzyme expression in liver, regulation of clock and circadian rhythm, and lymph node development [17]. Multiple aspect functions of RORγt give it the challenge as a therapeutic target, as many side effects will be invoked in this pathway. To get a T cell specific drug would resolve this dilemma. In this study, we found these four candidates exhibited suppression on RORγt activity in T cell derived cell-Jurkats, rather than in 293 T cells, which provided the potential of cell type specificity. This specificity between two cell lines will provide the possibility to develop T cell specific drugs from these candidate compounds.

Although RORγt plays a crucial role in Th17 differentiation and IL-17A production, studies have determined that it has no significant impact on IL-17A promoter activity [33]. Recent studies showed that the regulation of IL-17A and IL-17 F expression by RORγt involved CNS2 regulatory region at the il17-il17f locus [34]. It suggests that IL-17A and IL-17 F are direct targets of RORγt transcriptional regulation by binding to CNS2 region. In this study, we also found four candidate compounds displayed ~50 % inhibitory effect on IL-17A expression, similar to that observed with SR1001 [22]. Apart from inhibition of IL-17A production, these candidates exhibited potently inhibitory activity on IL-17 F, which differs from SR1001. Results suggested that these compounds had different inhibitory activity against IL-17A and IL-17 F expression.

In addition, the EC_50_ values of compound 31 and compound 35 indicated that they have lower efficacy compared with other two candidates. Fortunately, compounds 29 and 36 had potently inhibitory activity and showed moderate efficacy in suppressing RORγt activity in our Gal4-reporter system. Furthermore, these two candidates also showed limited cytotoxic effects in wild-type Jurkat cells, with CC_50_ values >5 μM as determined by MTT assays. Nevertheless, there is still room for optimizing structures of these candidates to acquire higher efficacious antagonist in future studies.

## Conclusions

In the present study, we screen and identify four candidate compounds with potent antagonistic activities to RORγt. These compounds sufficiently inhibit Th17 differentiation and IL-17A and IL-17 F production with high T cell specificity and low cytotoxicity. Thus, these compounds could be lead candidates for developing drugs in the treatment of autoimmune diseases. However, further experiments are necessary to identify their effects in vivo.

## Abbreviations

HTS: High-throughput screening
RORγt: Retinoic acid receptor-related orphan gamma t
RORγt-LBD^+^ Jurkat cells: Jurkat-stable cells which express the reconstructed plasmid pBIND-RORγt-LBD-IRES-GFP and the reporter plasmid pGL4.31 (luc2P/GAL4UAS/Hygro)
RORγt-LBD^+^ 293 T cells: 293 T-stable cells which express the reconstructed plasmid pBIND-RORγt-LBD-IRES-GFP and the reporter plasmid pGL4.31 (luc2P/GAL4UAS/Hygro)

## References

[1] Chambon P (2005). The nuclear receptor superfamily: a personal retrospect on the first two decades. Mol Endocrinol.

[2] Jetten AM (2004). Recent advances in the mechanisms of action and physiological functions of the retinoid-related orphan receptors (RORs). Curr Drug Targets Inflamm Allergy.

[3] Solt LA, Burris TP (2012). Action of RORs and their ligands in (patho)physiology. Trends Endocrinol Metab.

[4] Ivanov II, McKenzie BS, Zhou L, Tadokoro CE, Lepelley A, Lafaille JJ (2006). The orphan nuclear receptor RORgammat directs the differentiation program of proinflammatory IL-17+ T helper cells. Cell.

[5] Sun Z, Unutmaz D, Zou YR, Sunshine MJ, Pierani A, Brenner-Morton S (2000). Requirement for RORgamma in thymocyte survival and lymphoid organ development. Science.

[6] Ruan Q, Kameswaran V, Zhang Y, Zheng S, Sun J, Wang J (2011). The Th17 immune response is controlled by the Rel-RORgamma-RORgamma T transcriptional axis. J Exp Med.

[7] Peelen E, Damoiseaux J, Smolders J, Knippenberg S, Menheere P, Tervaert JW (2011). Th17 expansion in MS patients is counterbalanced by an expanded CD39+ regulatory T cell population during remission but not during relapse. J Neuroimmunol.

[8] Smith AW, Doonan BP, Tyor WR, Abou-Fayssal N, Haque A, Banik NL (2011). Regulation of Th1/Th17 cytokines and IDO gene expression by inhibition of calpain in PBMCs from MS patients. J Neuroimmunol.

[9] Li Y, Jiang L, Zhang S, Yin L, Ma L, He D (2012). Methotrexate attenuates the Th17/IL-17 levels in peripheral blood mononuclear cells from healthy individuals and RA patients. Rheumatol Int.

[10] Murdaca G, Colombo BM, Puppo F (2011). The role of Th17 lymphocytes in the autoimmune and chronic inflammatory diseases. Intern Emerg Med.

[11] Leppkes M, Becker C, Ivanov II, Hirth S, Wirtz S, Neufert C (2009). RORgamma-expressing Th17 cells induce murine chronic intestinal inflammation via redundant effects of IL-17A and IL-17 F. Gastroenterology.

[12] Aranami T, Yamamura T (2008). Th17 Cells and autoimmune encephalomyelitis (EAE/MS). Allergol Int.

[13] Shabgah AG, Fattahi E, Shahneh FZ (2014). Interleukin-17 in human inflammatory diseases. Postepy Dermatol Alergol.

[14] Mease PJ (2015). Inhibition of interleukin-17, interleukin-23 and the TH17 cell pathway in the treatment of psoriatic arthritis and psoriasis. Curr Opin Rheumatol.

[15] Yang J, Sundrud MS, Skepner J, Yamagata T (2014). Targeting Th17 cells in autoimmune diseases. Trends Pharmacol Sci.

[16] Xie H, Sadim MS, Sun Z (2005). RORgammat recruits steroid receptor coactivators to ensure thymocyte survival. J Immunol.

[17] Jetten AM (2009). Retinoid-related orphan receptors (RORs): critical roles in development, immunity, circadian rhythm, and cellular metabolism. Nucl Recept Signal.

[18] Onate SA, Boonyaratanakornkit V, Spencer TE, Tsai SY, Tsai MJ, Edwards DP (1998). The steroid receptor coactivator-1 contains multiple receptor interacting and activation domains that cooperatively enhance the activation function 1 (AF1) and AF2 domains of steroid receptors. J Biol Chem.

[19] Glass CK, Rosenfeld MG (2000). The coregulator exchange in transcriptional functions of nuclear receptors. Genes Dev.

[20] Fujita-Sato S, Ito S, Isobe T, Ohyama T, Wakabayashi K, Morishita K (2011). Structural basis of digoxin that antagonizes RORgamma t receptor activity and suppresses Th17 cell differentiation and interleukin (IL)-17 production. J Biol Chem.

[21] Huh JR, Leung MW, Huang P, Ryan DA, Krout MR, Malapaka RR (2011). Digoxin and its derivatives suppress TH17 cell differentiation by antagonizing RORgammat activity. Nature.

[22] Solt LA, Kumar N, Nuhant P, Wang Y, Lauer JL, Liu J (2011). Suppression of TH17 differentiation and autoimmunity by a synthetic ROR ligand. Nature.

[23] Solt LA, Kumar N, He Y, Kamenecka TM, Griffin PR, Burris TP (2012). Identification of a selective RORgamma ligand that suppresses T(H)17 cells and stimulates T regulatory cells. ACS Chem Biol.

[24] Kumar N, Kojetin DJ, Solt LA, Kumar KG, Nuhant P, Duckett DR (2011). Identification of SR3335 (ML-176): a synthetic RORalpha selective inverse agonist. ACS Chem Biol.

[25] Kumar N, Lyda B, Chang MR, Lauer JL, Solt LA, Burris TP (2012). Identification of SR2211: a potent synthetic RORgamma-selective modulator. ACS Chem Biol.

[26] Huang Z, Wang R, Xie H, Shang W, Manicassamy S, Sun Z (2008). Stabilized beta-catenin potentiates Fas-mediated T cell apoptosis. J Immunol.

[27] Bennukul K, Numkliang S, Leardkamolkarn V (2014). Melatonin attenuates cisplatin-induced HepG2 cell death via the regulation of mTOR and ERCC1 expressions. World J Hepatol.

[28] Korn T, Bettelli E, Oukka M, Kuchroo VK (2009). IL-17 and Th17 Cells. Annu Rev Immunol.

[29] Miossec P (2009). IL-17 and Th17 cells in human inflammatory diseases. Microbes Infect.

[30] Mongrain V, Ruan X, Dardente H, Fortier EE, Cermakian N (2008). Clock-dependent and independent transcriptional control of the two isoforms from the mouse Rorgamma gene. Genes Cells.

[31] Muhlbauer E, Bazwinsky-Wutschke I, Wolgast S, Labucay K, Peschke E (2013). Differential and day-time dependent expression of nuclear receptors RORalpha, RORbeta, RORgamma and RXRalpha in the rodent pancreas and islet. Mol Cell Endocrinol.

[32] Chen Y, Coulter S, Jetten AM, Goldstein JA (2009). Identification of human CYP2C8 as a retinoid-related orphan nuclear receptor target gene. J Pharmacol Exp Ther.

[33] Crome SQ, Wang AY, Kang CY, Levings MK (2009). The role of retinoic acid-related orphan receptor variant 2 and IL-17 in the development and function of human CD4+ T cells. Eur J Immunol.

[34] Yang XO, Chang SH, Park H, Nurieva R, Shah B, Acero L (2008). Regulation of inflammatory responses by IL-17 F. J Exp Med.

